# The security implications of using feminist methodologies to study gender-based violence in Yemen

**DOI:** 10.3389/frma.2024.1333266

**Published:** 2024-11-18

**Authors:** Sawsan Al-Refaei

**Affiliations:** Independent Researcher, Ottawa, ON, Canada

**Keywords:** gender, violence, methodology, risk, feminist, Yemen, researcher, security

## Abstract

**Introduction:**

This paper examines the political and security implications of gender-based violence (GBV) research in Yemen during the period (2019–2023). As various radical groups are gaining power over Yemeni land, radical views toward women and gender equity and equality shape the experiences of GBV survivors, practitioners, and researchers in the North of Yemen. Policing Houthi ideologies in Yemen have curtailed GBV research and subjected feminist research to myriad risks. If this situation continues, experiences of women and girls in Yemen will not be captured by research. Their stories and needs will not be captured by humanitarian and peace-building actors.

**Methodology:**

Findings of this study are based on primary data from key informant interviews with 25 GBV researchers actively engaged in Yemen. Sampling followed the snowballing technique.

**Results:**

The findings examine political and security power dynamics after the Houthi radical group took control in the north of Yemen, and implications on GBV research design. Anti-feminist ideologies coupled with extreme security measures have impacted quality of GBV study methodologies as well as researchers' safety and mobility. Donors of previously established GBV programs and research were harassed to change research topics or lose permission to speak to local communities or collect data from aid beneficiaries. Researchers who do not follow new rules of engagement with the community are detained, harassed and their devices and databases are confiscated. Terms like “gender” and “GBV” are not deemed acceptable as these are western concepts that do not align with new community values. The findings highlight the need to use conflict-sensitivity and Do No Harm principles in settings where GBV work is scrutinized. It also challenges the orthodox definition of “GBV evidence” and explores the ethical implications of the use of alternative means to collecting data. Findings also provide insight into valuable alternative methodologies that allow local and national researchers to continue studying GBV in conflict impacted areas without exposing themselves to actual or perceived risk. The paper proposes concrete approaches that can mitigate political and security risk on both researchers and GBV survivors.

## 1 Introduction

### 1.1 Gender-based violence in Yemen

Amidst protracted crisis, gender-based violence has become endemic in Yemen with more than 6.36 million women and girls at heightened risk of its various forms, including harmful traditional practices (UNFPA, 2021). Gender-based violence's (GBV) roots in Yemen can be traced back to periods predating the contemporary conflict. It has disproportionately impacted women and girls from minority racial groups, migrants, refugees and asylum seekers (OXFAM, 2020). Around 30 per cent of girls in Yemen are married before the age of 18 (UNICEF, 2022). Rising divorce, temporary marriages, and inflation [(International Rescue Committee (IRC), 2023)] have left more women and girls and female-headed households disproportionately vulnerable [(International Rescue Committee (IRC), 2023)].

GBV in the form of honor-related homicides, female genital mutilation, coerced and child marriage, sexual harassment and rape, and marital rape have risen (ACAP, 2023). Perpetrators, encompassing family members, kin, and community individuals, exploit the vulnerability of societal safeguard systems, thereby amplifying the gravity and breadth of their offenses. Furthermore, women are frequently subjected to physical maltreatment and torture (Al-Ammar et al., 2019) with the intent of dissuading them from public participation. Economic coercion against women manifests as infringement of rights and resources, educational deprivation, income regulation, and inheritance denial. Displacement can put women and girls at higher risk, and the absence of male family members who are fighting or earning a living away from their homes, denies female family members protections they normally have (UNFPA, 2022).

The magnitude and range of these crimes have been accentuated by the triad of poverty, the repercussions of the COVID-19 pandemic (Peace Track Initiative, 2022), and climate change. The situation has become more dire due to the disintegration of judicial and law enforcement institutions, lack of reliable GBV referral and data systems, unsupported and dwindling GBV services and brain drain of qualified GBV frontline staff. Reports show that less than five per cent of health facilities provide clinical management of rape, and 90 per cent of rural areas lack GBV services (UNFPA, 2023).

The Houthis, a militant group based on radical and anti-feminist religious ideologies (Sarhan et al., 2022), took over the Yemeni capital Sanaa in September 2014 and seized control over much of northern Yemen by 2016 (Wilson Center, 2022). The group developed a highly repressive security system and reports directly to radical leadership unrecognized internationally. Their ideologies carry discernible bias against initiatives anchored in gender development or feminist theoretical frameworks (Middle East Institute, 2021).

The ideological orientation of Houthi armed factions, coupled with the ascendancy of radical religious perspectives, has further relegated women to the domestic sphere with heightened mobility constraints (Relief Web, 2023), necessitating the accompaniment of a male kin (mahram)^1^ for both local and cross-country journeys. This has resulted in economic burdens and diminished access to public amenities. It has also impinged upon opportunities for employment and accessing vital services, like food aid (OCHA Yemen, 2015).

Disturbingly, Houthis have progressively been putting measures in place to counteract the burgeoning enthusiasm and prowess of women in societal mobilization since the 2011 uprisings (The Guardian, 2011). Public Houthi narratives have depicted women in the public sphere as immoral (United Nations, 2018) using derogatory terms, to delegitimize and curb their public presence mainly in political and civil spaces. This has led to the curtail of civil and political work of women activists and in severe cases has led to marked constraints placed on women civil leaders and activists. Extreme cases include arbitrary detainment, kidnapping and imprisonment of women (United Nations, 2021). Furthermore, female humanitarian personnel including female GBV researchers have been systematically targeted. Sporadic GBV assaults, which were once isolated incidents, have morphed into orchestrated onslaughts, disparaging women civil society leaders and humanitarian workers (United Nations, 2021).

### 1.2 Gender tabooing ideology in Northern Yemen

In recognition of the strategic potential of international aid, Houthi leaders established a body named the Supreme Council for the Management and Coordination of Humanitarian Affairs and International Cooperation (SCMCHA). This official body is mandated to oversee all stages of humanitarian work from needs determination to coordination of funds, reviewing implementation reports and corresponding with donors. ^2^ With SCMCHA monopolizing humanitarian efforts in Houthi-controlled regions, any civil society entities engaged in aid must comply and face comprehensive oversight (Naser, 2022). Houthi authorities used SCMCHA processes to disapprove studies that may implicate them or hold them accountable to failure to prevent and respond to GBV. This is more prominent in areas where GBV has been documented in internally displaced persons (IDP) camps, including increases in the number of GBV cases (Saleh, 2020).

Beginning in 2019, SCMSHA has increasingly discouraged initiatives addressing gender disparities, including gender analyses, assessments, and gender-focused research. Their public statements have insinuated that humanitarian efforts, specifically GBV data collection, have intelligence gathering purposes and are not designed to assess impacts of conflict and effectiveness of aid. The Houthi ideology views terms like “gender,” “women's rights,” and “women's empowerment” as clashing with national and Islamic values (Wilson Center, 2023). Data on the impact of aid on women or forms of violence targeting women and girls (whether domestic or at the community level, excluding airstrikes) might uncover the deteriorating conditions of women and girls and accordingly could bolster the advocacy efforts of women's groups and activists. This data could be utilized to push for relaxed restrictions on women in public spaces or demand greater female representation within the Houthi administration (Al-Refaei, 2022).

This study examines the impact of Houthi state de-facto regulations to curtail GBV research. It examines both the impact on Yemen women and girls and on researchers who continue their GBV work under strict political and security risks.

## 2 Methodology

Findings of this study are based on primary data from key informant interviews with 25 GBV researchers actively engaged in Yemen. The author interviewed 20 women and three men working as researchers and data collectors who currently reside in 5 Yemeni governorates. The uthor also interviewed two women research team leaders who live in the diaspora outside Yemen but supervise field teams working in Yemen. Sampling followed the snowballing technique. The author selected two GBV researchers who have published a GBV study online, one male and one female, and asked them to obtain permission from two others prior to disclosing their contact information. Using this method, the author was able to conduct 25 in-depth interviews in Arabic via Google Meet and Microsoft Teams. Interviews were not recorded or digitally typed based on the request of interviewees.

The interview guide focused on three main themes, that formed the basis of the analysis:

- Ways in which researchers adopted their methodologies to the strict political and security measures enforced by de-facto state actors in northern Yemen.
- Risks facing GBV researchers in Yemen, using a gender lens.
- Recommendations on alternative GBV research methodologies to fit the current context in Yemen.^3^

GBV researchers are few in Yemen. For that reason, information, quotes, and data that could help identify interviewees are not shared for their security and safety, including demographic and geographical location data.

## 3 Results

### 3.1 Structural barriers to GBV research

All interviewed researchers highlighted that Houthis have introduced very strict limitations on gender-focused research. A woman researcher pointed out that terms like gender and “violence against women are often met with suspicion, linking them to a West-based conspiracy.”^4^ She added, “They see feminist research as against their national and religious beliefs.”

All respondents stated that SCMCHA is a body established to collate intelligence to high-ranking Houthi officials about work of civil society organizations, enforce control over both local and international humanitarian actors, and siphon off portions of international humanitarian aid funding. SCMSHA, when they officially portray themselves as partners and coordinators, in reality they are perceived by the research community as “data police.”^5^

A field researcher from one rural area described how SCMCHA “conducts meticulous examination of research objectives, methodology, and scope prior to issuing an official authorization to commence fieldwork.” “Approval is not guaranteed,” stated one researcher in Sana'a. “Our team have waited for more than 5 months to receive permit for a gender analysis study, and we never got it. The donor was no longer able to wait and so the project was canceled.”^6^

The complex process of obtaining approvals has discouraged stakeholders, mainly UN agencies and donors to invest in GBV research. According to a local man data collector in the north, “the linguistic *tabooing*^7^ serves as an ideological instrument: it not only marginalizes issues pertinent to women but concurrently casts them in a problematic and dubious light.”^8^

SCMSHA, in collaboration with affiliated security entities, retains the authority to sanction, amend, or reject research proposals, and the right to ask for amendments to methodology and sampling. They possess the discretion to excise or alter research questions and modify the linguistic structure of data collection instruments as deemed appropriate. Furthermore, “they hold the prerogative to adjust the geographical breadth of research, modify the attributes of the intended study population, and recalibrate safeguarding protocols based on their evaluative judgment.”^9^ Permits of humanitarian research, for example, are provided with the precondition of omitting direct reference to gender or GBV. The term “violence,” when addressed within the research, is contextually defined in terms of physical aggression against the state or nation, rather than as a societal issue. Permits are also granted for very short durations to limit the comprehensiveness of the data collected. Geographical regions that have recently garnered media attention under Houthi governance are typically off-limits for research. “Studies aiming to address vulnerable or marginalized populations such as IDPs often face rejection or intensified scrutiny.”^10^ In some cases, authorization is contingent upon the inclusion of a SCMSHA representative within the research team, or, at a minimum, allowing said representative to accompany the team during all field activities. “This may involve training of one of their officials either as a data collector or a field supervisor to ensure more stringent data oversight.^11^ A woman GBV expert validated (Vuylsteke, 2021) that“gender assessment research teams are frequently accompanied by Houthi authorities who introduce themselves as researchers, which puts studied populations at huge risk.”

### 3.2 Adapting GBV research methodologies to the context

Researchers engaging in GBV topics are compelled to “excise the term ‘gender' from all data collection instruments, narratives, or ensuing reports; a failure to do so results in the discontinuation or non-publication of the research.” A distinguished GBV scholar based in Yemen articulated, “I received an explicit directive to eliminate the term ‘gender' from the reference list of my ongoing study.” The constriction of terminological usage has expanded to encompass all subjects related to women, even extending to topics such as lactation and reproductive health. “The term ‘health' has become the designated nomenclature.”^12^ Several interviewed researchers named an example of the severe backlash faced by university professors in a renowned university upon incorporating a women and gender development course into their curriculum.^13^ “The University was accused of normalizing non-Islamic values and promoting moral disintegration of Yemeni society,” one female researcher added. Prior to the onset of the 2015 conflict, the Yemeni government employed quantitative, qualitative, and mixed method approaches to study GBV within the context of national assessments. She added, “The National Health and Demographic Survey^14^ and the General Population Housing and Establishment Census were progressively integrating a women and gender lens.” Between 2015 and 2019, a series of seminal studies examining the gendered ramifications of the Yemeni conflict and specific facets of GBV were undertaken. “The most recent comprehensive GBV research, executed in 2019, harnessed an array of data collection strategies, encompassing focus group discussions, in-depth informant dialogues, and case studies” explained a senior staff in a major research center in Sana'a.^15^ From the year 2020, “the acquisition and dissemination of primary GBV data encountered formidable obstacles. As randomized quantitative approaches may bring in unsurmountable complexity to research projects under such political environment, researchers chose to rely on qualitative techniques and chose to continue executing GBV research without permits.”

Researchers started questioning some of the techniques they have traditionally used. “Using Focus Group Discussions (FGDs) enables women, girls, and even men to share nuances and dynamics of GBV compared to individual interviews or surveys,” articulated a seasoned female researcher specializing in GBV in IDP contexts. Under the new Houthi regulations, the roster of FGD participants must be declared and approved by authorities subjecting participants and researchers to risk. In numerous instances, Houthi representatives stipulate that they either handpick the FGD participants or be present during the sessions. An expert in FGD methodologies remarked,^16^ “In earlier times of Houthis, we would present fabricated FGD questionnaires just to get approval; such maneuvers are now untenable.”

Interviewed researchers said that semi-structured interviews have emerged as the singular viable choice for data collection on GBV, primarily due to their potential for discretion in the absence of official authorization. Interviews are almost always semi-structured, because of the impracticality and risks of in-depth interviews.^17^ While semi-structured interviews might not yield comprehensive data, several male researchers shared that the format's inherent flexibility permits them to pivot topics if they perceive potential threats or sense any surveillance. For delving into specific aspects of GBV, especially sexual harassment and assault, semi-structured interviews are not the most efficacious compared to unstructured ones, especially within precarious settings like displacement camps or detention centers. “Short interviews tend to be truncated, depriving researchers of the requisite duration to foster trust with respondents and to meticulously explore the intricate facets of GBV”^18^ stated a human rights monitor.

For safety considerations, these interviews are predominantly convened within the confines of a participant's residence by female researchers. “Occasionally, we solicit participants to rendezvous in public venues, spaces where the presence of women and girls is deemed ordinary, in a bid to deflect any undue attention from local Houthi officials.”^19^

When Houthi local leaders heard that GBV studies were secretly being done without permission, they started watching group meetings or other public events even more closely. One person said, “Houthi officials reward or punish locals to get them to report on any research happening.”^20^ Thus, researchers have ventured into employing telephonic semi-structured interviews, typically constrained to a duration of 30–40 min. One male scholar, specializing in human rights violations, articulated the inherent challenges in corroborating identities and maintaining a consistent conversational flow during such telephonic engagements. “Interlocutors frequently manifest signs of discomfort or display a reluctance to prolong the call,” he noted, adding that culturally, extended telephonic interactions with stranger males are not culturally endorsed.^21^

Researchers have also trialed text-based interviews, leveraging platforms like WhatsApp. This approach entails dispatching succinct questions to respondents, who subsequently possess the discretion to either orally record their feedback or articulate it in written form. “Researchers ensure through close follow up that conversations are deleted” explained multiple interviewed researchers, however risk on interviewees remain.

### 3.3 Concerns around the adapted methodologies

Researchers expressed their concerns around four key aspects: bias, representativeness, quality, and confidentiality.

Sample selection in the context described above leads to selection bias. Researchers are often limited to interacting with community members they, or their associates, deem trustworthy and those equipped with requisite technology like mobile phones and internet connectivity. “We often miss the most vulnerable women and girls” stated a women field researcher.^22^ In a similar vein, online interactions utilizing platforms like Zoom and Skype predominantly involve participants from relatively privileged educational and economic backgrounds, including civil society representatives, members of the Yemeni diaspora, and political dignitaries. A researcher explained, “the internet penetration rate is very low in Yemen (Datareportal, 2022), our research predominantly captures the perspectives of the elite. We expect response bias from local communities, [….] they anticipate Houthi surveillance, and may provide inaccurate data in fear of being denied humanitarian assistance or being reported to the Houthi authorities.”

Houthis have imposed constraints on the work and mobility of researchers renowned for their feminist orientation. GBV researchers once pooled from qualified academia, now compose of a diverse range of individuals—from human development facilitators and humanitarian aid workers to former or active military personnel. Despite potential shortcomings in GBV sensitivity, these local agents have superior accessibility to women and local communities, incurring fewer risks in the process. The inexperience of research personnel inevitably raises concerns about the potential compromise in data quality and adherence to research ethics.

In detention facilities and IDP camps, researchers have exhibited reluctance to possess any paper interview guides or even notebooks. A female monitor of women prisoner conditions elaborated, “we memorize a set of 5–6 questions and proceed with the interviews.” She added, “in the rare cases when we can take notes or record interviews without being noticed, we do not adhere necessarily to consent protocols.”

At times, ensuring the safety of interviewees necessitates a compromise on privacy. As one researcher pointed out, “the most secure venues for conducting interviews with women are typically places where their presence is anticipated, such as mosques, women's centers, and domestic settings. These locations often offer the least amount of privacy.”^23^

These trade-offs between access and safety are made by individuals undertaking research, as noted above, who may not have the necessary training in research ethics. While data collection is important, a core principle of research ethics is beneficence, which means researchers must act in ways that benefit research populations while protecting their safety and welfare. The decision to prioritize collecting data over obtaining informed consent, for example, violates research ethics and calls into question the validity of the findings. These decisions are also made on behalf of a population that is not necessarily well-informed about the risks entailed. There may also be risks for the researchers themselves, as detailed in the next section. Overall, the difficult conditions in Yemen do not provide an excuse to ignore GBV research ethics (PATH, 2005) which places an imperative to put the safety of research subjects first.

### 3.4 Chronic and new risks facing GBV researchers

- Feminist researchers face myriad challenges, including arbitrary detentions, data confiscations, and movement restrictions. One researcher recounted, “I was detained for 17-days, some of which I spent in solitary confinement, because military officials found research documents on my laptop during search.”^24^ Detained researchers can face accusations ranging from unauthorized data collection to purported acts of national treason. Four interviewees described checkpoints within or between cities as important risk areas. “In the event of laptop or mobile confiscations, military personnel will attempt to access personal and professional information stored within, including identifiers, photographs, correspondence, and social media accounts. This breach of privacy not only threatens the researcher but also their families, potentially exposing them to GBV, exploitation, or blackmail. This also breaches the confidentiality of the GBV data that may not have been protected adequately,” a male researcher. explained. For that reason, many researchers conceal their true identities or professions, especially at checkpoints. One researcher^25^ noted, “I often travel as a coffee trader, which facilitates my movement and allows me to gather data and train field personnel.”
- The pervasive environment instills a sense of fear among researchers as they can be reported any time. Recounting an unsettling incident, a female researcher mentioned, “Minutes into a focus group discussion I was facilitating, one participant warned me to stop asking questions, or she would call her brother working as military personnel, to intervene.” Multiple researchers that their study population do not fully grasp the value of their work and view them instead as harbingers of risk and unease with no perceivable benefits. There have been instances where researchers, particularly females, were reported to local Houthi groups either in hopes of receiving food aid or to foster a sense of trust with the militia.

#### 3.4.1 Gendered risks on researchers

While tribal norms^26^ traditionally shield women from harm during armed conflict (UN Women, 2019), these conventions haven't safeguarded Yemeni feminist researchers from violence (US Department of State, 2022). As one highlighted, “Some female researchers probing sensitive areas like GBV within prison settings have faced detentions extending up to 5 days.”^27^ While male researchers are not exempt from these transgressions, the societal repercussions for detained female researchers are more pronounced due to social stigmas. “There have been cases of women who were detained and subsequently divorced by their husbands.”^28^

One researcher mentioned, “women researchers often lack the social means to obtain free legal representation or assistance. In certain situations, they might be reluctant to alert her family.”^29^ Women researchers tend to be released more promptly from detentions, and any personal searches are typically conducted by female security personnel. However, the seizure of their data, inclusive of personal information, leaves them vulnerable to sexual exploitation, blackmail, and other forms of harassment at the hands of male security officials.

Women researchers have occasionally adopted the strategy of wearing the niqab (face cover) to become less identifiable by security forces, particularly during household-to-household interviews. Like other women in northern Yemen, they face travel constraints unless accompanied by a male relative, “*mahram*,” or in possession of documented consent from their mahram or from a local neighborhood supervisor. “They cannot hire cars independently, might be denied service by bus drivers, and face challenges in securing hotel accommodations without male company” stated a woman researcher.

Similar constraints apply to women aspiring to travel by air in acquiring national IDs. Such barriers have impeded the fieldwork of female researchers, compelling many to abandon their professional pursuits. This not only results in a loss of critical income for their households but also constricts the breadth of GBV-focused research. Moreover, these limitations have thwarted Yemeni female researchers from showcasing their GBV studies on international platforms and from acquainting themselves with relevant research from other global contexts.

In the context of a weakened rule of law and the dissolution of numerous GBV organizations in Yemen (Yemen Policy Center, 2022) women researchers grapple with limited recourse to protection and remedy of GBV health impacts. “The absence of legal and security protection measures in my contract has left me in a state of perpetual anxiety. I have been chronically impacted by nightmares and severe psychological symptoms, yet my contractual obligations as a researcher don't encompass provisions for psychosocial support or health insurance,” a researcher shared.^30^

Women researchers with the ability to disseminate their GBV findings internationally often face online harassment and bullying. A young Yemeni female researcher who shed light on GBV among marginalized groups recounted, “Antagonists of my work reached out to the institution sponsoring my postgraduate studies, urging them to withdraw their financial support.”^31^

Regrettably, the response of International Organizations (IOs), inclusive of United Nations agencies, to the pressing needs of GBV researchers (Gupta et al., 2023)^32^ was less than expected. The concessions these institutions have made in compliance with Houthi directives are considerable. “They agreed to discontinue previously planned GBV research,” a female researcher in close relationship with the United Nations operations in Yemen explained. “The UN turned a blind eye to the newly imposed regulations that curtail the freedoms and mobility of women researchers.” Additionally, they have made modifications to their third-party monitoring frameworks, significantly diluting the emphasis on gender and GBV indicators. Such oversights inadvertently facilitate negative consequences, including the unintended role of aid distribution in exacerbating GBV.^33^

Female researchers highlighted the reticence of IOs to accommodate the expenses associated with ensuring safe travel routes, addressing legal fees, travel costs of male companions, or packages tailored to address psychological trauma. Researchers are required to continue to deliver with no recovery time granted. IOs contract researchers on a short-term basis and do not interfere when they get caught by Houthis to maintain political relations. “It is predominantly our local connections that come to our rescue in dangerous situations; be it familiar local police officers, political affiliates, or friends residing in those specific regions,” a female researcher stated.^34^

Constraints on women's freedoms, especially those impacting qualified female gender researchers and data collectors, are expected to result in a decline in feminist narratives on trends of GBV. The increasing risks on women researchers is also leading to “brain drain of feminist researchers” as many researchers chose to “leave northern Yemen, leave this area of work, or leave Yemen altogether,”^35^ an international expert added.

#### 3.4.2 Risks on researched populations

An interviewed female international researcher shared, “against common perception, some women, girls as well as men are ready to speak about sexual, physical and other forms of violence. They may be hesitant at first, but they want the violent reality to change. When researchers speak the local dialect and seem trustworthy, community members share information deeper and wider in scope than we ever expect.” She added, “communities want to participate, it is fear from Houthis, and fear of being shamed and isolated by their community that makes them refuse to give interviews.”^36^

Communities, especially women bearing the brunt of hunger and displacement, fear losing aid. While most of the researchers interviewed argue that the risks encountered when doing GBV research are “worth taking” for the potential long-term benefits to the safety of women and girls in the long run, there is no evidence that the researched population shares that view or understands the risks sufficiently to provide their informed consent, which is a necessary ethical component of all research. One researcher interviewed expressed disappointment that “some Yemeni communities have fallen for the pervasive narrative of the media and local religious leaders that considers gender-focused initiatives un-Islamic,” implying that interviewees are too afraid to speak the truth.^37^

The level of need is such that some researched populations may decide to take on the risk of participating if some form of compensation is offered. As GBV research often targets the most vulnerable and marginalized populations, respondents from these groups have been vocal about their need for compensation, which is not always budgeted for in research projects but which also calls into question whether consent is freely given in these cases, or whether this is in effect a form of coercion. “Certain participants are motivated to disclose information in exchange for tangible benefits like food baskets, cash, or services. The majority if not all research projects do not budget for compensation of the researched population,” a male researcher shared. “This is justified by the fact that compensation may bias research results and challenge the ethical imperative of freely given consent to participate.”^38^

### 3.5 Alternative data collection methodologies with ethical considerations

“If we want to continue collecting GBV data, there is a need for us to be innovative,” a woman researcher stated. She suggested “It is time to support a new generation of data collectors particularly young women, who demonstrate a keen aptitude for learning and are deeply embedded within local social structures. They can effectively gather GBV data due to their entrenched position within the community, offering both motivation and a layer of protection.” A male researcher stated that historically, professionals such as midwives, illiteracy teachers, nurses, and local farmers/entrepreneurs have been used with little training to collect health related data including sensitive GBV data such as data on child marriage and female genital mutilation. Their positions in the community naturally grant them unobstructed access to local households, IDP camps, and diverse segments of the female population. Other interviewed researchers imagined new models such as “researcher families.”^39^ She explained that a family unit, consisting of related adults from different genders and youth, undertakes data collection collectively rather than as isolated individuals. While women and girls can be interviewed with women researchers only, male relatives of the researcher can serve as research companions, data analysts, and report writers. Interviewed researchers did not share the risks attached to these models, or how they will ensure data confidentiality and research subject safety.

Young researchers find that social media can serve as alternative channels to conduct polls and collect qualitative data.^40^ A man researcher shared. “Youth demographic remains an underutilized resource, especially when considering data collection amongst their peers, who might be less susceptible to official scrutiny.” “Researchers can potentially harness AI-integrated platforms to collate feedback on GBV, deploying a consistent set of questions. This approach would facilitate anonymous contributions, shielding both the researcher and the participant's identities,” he added. It remains unclear how the proposed alternative methods of data collection, will eliminate risk to research subjects, and how data can be cross examined for quality and non-bias.

## 4 Discussion

### 4.1 Redefining data collectors: an analysis of research ethics

During heightened surveillance and socio-political scrutiny, establishing trust between researchers and local communities becomes paramount. Prioritizing the development of robust relationships between local populations, community leaders, and local researchers is imperative to minimize risks on both data collectors and the subjects of research. Therefore, localization of data collection and support of local researchers through provision of protection and an enabling environment is crucial for their work to continue. Nevertheless, both women and men GBV researchers in northern Yemen, have adapted to the political and security limitations of their work in ways that jeopardize the researched population's safety and agency. While researchers have relied on local connections to recruit study subjects and also to secure spaces for interviews, there is a potential for selection bias by local brokers. Moving from the notion of research elite to “research frontliners” is posited as a pivotal shift for the continuity of gender and GBV studies. Local professionals like teachers and midwives may carry inherent ability to interact with a spectrum of women and girls, coupled with adept communication skills, but there is little evidence that they will be able to acquire adequate skills on trauma-informed data collection and follow do no harm principles given the security context. Engaging local professionals also requires their own interest in being engaged and trained, and in taking on the risks associated with such research, as outlined above. It may also carry the unintended negative effect of undermining the important societal roles they already serve, as trusted community members providing reproductive healthcare or education.

### 4.2 Safety of researchers and the researched

The safety and security of both researchers and the researched must be prioritized at all times (Ellsberg and Heise, 2005). To do this, it is pivotal that humanitarian budgets recognize and allocate resources for the safety of GBV researchers. Provisions should encompass safe transportation, legal aid, and psychosocial support costs, ensuring a holistic approach to their protection. During the planning stage, research locations should be avoided where participants or researchers must travel through high-risk areas if appropriate safety measures are unavailable. Findings from interviews reveal that adequate risk assessments and mitigations were not put in place.

Numerous organizations operating in settings such as Yemen and other emergency scenarios have instituted protection protocols and operational standards for their staff. While a comprehensive assessment of their efficacy and implementation is beyond the scope of this discussion, a recurring theme is that these standards do not respond to the emerging challenges faced by researchers, particularly in roles related to GBV research. Findings from interviews highlight the discrepancy between protection services offered to staff vs. researchers on short-term contracts. These findings are serious as they may indicate that GBV researchers are contracted short-term to evade political and financial accountability on the humanitarian organizations that hire them.

There is an imperative to extend protection guidelines and additional training and re-training (Ellsberg and Heise, 2005) to encompass all researchers, data collectors, and advocates even those not permanently hired. Tailored operational standards are required, ensuring adherence to the “do-no-harm” principle during the recruitment and deployment of these experts to the field. For beneficiaries of aid, who are also asked about GBV, there is a need to use utmost caution to avoid the identification of individuals who may be targeted later for their participation in the research or service delivery. This will require new data safety protocols, inclusive of hard copy and soft copy data, to ensure the personal information of research subjects is never linked to data collected from them, in case researchers' data was confiscated.

Once the research team has left, a follow-up process should be in place to monitor any unexpected consequences such as retaliation against women from male partners or others. Logistical and safety issues should be accounted for in the design and budget. At a basic design level, urban and rural variations of travel safety should be assessed. Findings from interviews show that data can be lost or confiscated because of multiple checkpoints and limited safe options for transportation. Safer transport means should be made available for researchers by the organizations that contract them. This includes provision of safety for women researchers and budgeting for a male companion if that will make their job easier and safer. It is pivotal that humanitarian budgets recognize and allocate resources for the safety of GBV researchers. Provisions should encompass safe transportation, legal aid, and psychosocial support costs, ensuring a holistic approach to their protection.

Furthermore, international organizations and donors must shoulder a portion of the political and security implications associated with persevering with GBV research. This entails transitioning from a narrative of compliance to one that confronts and challenges the emergent constraints, particularly those inhibiting women's mobility.

There are studies (Pitter, 2016; Ali, 2023) that highlighted women professional such as midwives can play a role in screening for GBV. These studies can guide further discussion around collection of GBV data through non-standard research personnel. This discussion needs to put safety of both female health professionals and researched subjects and ethical considerations at the forefront. Exploration of using social media on data collection should be highly scrutinized for quality and potential of different forms of data bias as shown in previous studies (Srivastava and Mishra, 2023; Reveilhac et al., 2022).

## 5 Conclusion

Despite the challenges, conducting research in conflict zones is crucial to enhance our understanding of various interventions and their outcomes, as well as to highlight the struggles of affected populations. Failing to pursue this research can increase their vulnerability and foster complacency among those responsible for or contributing to their plight.

There is available evidence (Hossain and McAlpine, 2017; McAlpine et al., 2020; Sikweyiya and Jewkes, 2011) on the key challenges and considerations that contribute to the broader discourse on conducting sensitive and critical GBV research in challenging environments. Studies that explore the political and security barriers that impede GBV data collection in insecure and highly policed environments, such as Northern Yemen, are scarce.

The findings of this research align with studies that highlight the necessity of methodologies that are adaptable to the constraints of safety, security, and logistical complexities inherent in fragile and acute crisis settings (Hossain and McAlpine, 2017). Findings here align with earlier findings on the paramount importance of safety and ethical considerations in GBV research, both for researchers and those they are researching (Thomas et al., 2022). It also sheds light on caveats of privacy, confidentiality, and anonymity as critical protective aspects for participants from ongoing risks and community stigma (TRUST, 2018; Madhani et al., 2014).

Existing literature emphasizes the unique and ongoing challenges highlighted in this paper, including 493 the need for innovative problem-solving strategies and flexibility in research methods to address 494 urgent crisis needs. For example, the use of remote data collection tools as a response to challenges come with their own set of methodological and ethical implications (Vahedi et al., 2023).

This paper shares the reality faced by GBV researchers, many of them women, in the face of increasing repression and hostility in Yemen. While there is a desire to adapt models of data collection to more effectively surpass the security and political limitations present, these call into question whether the benefit would outweigh the risks for researched populations, and for the researchers themselves. It is recommended that researchers and organizations, including humanitarian actors, contracting local researchers in Yemen, take into account the available ethical guidance when developing research plans and designing methods to be used, and proactively monitor for unintended harms throughout the research process. Local researchers have an important role to play in these discussions, as do participatory data collection.

## Data Availability

The datasets generated and analyzed by this article are not readily available due to security risks posed to the interviewed personnel. Following the Do-No-Harm principle, the data cannot be made available.

## Footnotes

^1^A Mahram, defined as in Islamic jurisprudence (fiqh) refers to a person with whom marriage is prohibited because of their close blood relationship to a woman.

^2^Supreme Council for the Management and Coordination of Humanitarian Affairs and International Cooperation(SCMCHA) Official Website.https://www.scmcha.org/en/about-us (accessed August 6, 2023).

^3^Feminist research methodologies move from the mainstream scientific methods, from only collecting data for objective purposes, towards gender sensitive data collection and analysis. Feminist methodologies aim to produce knowledge through ethical and political perspectives, which focus on the critique and overcoming of gender-blind scientific approach, in addition to the articulation of gender equality contents, concepts, conceptions, aims, objectives and outcomes.

^4^Interview with woman-former researcher with Women National Committee in Yemen.

^5^Interview with man-researcher contracted by several international humanitarian organizations in Yemen in areas of water and sanitation, health and civil society.

^6^Interview with woman -field officer of economic development project in rural Yemen.

^7^Interviewee provided the quote in Arabic using the word “” which in this context means tabooing.

^8^Interview with man- researcher working for local data and evaluation firm.

^9^ibid.

^10^Interview with woman- academic professor in University of Sana'a Yemen.

^11^ibid.

^12^Interview with woman- international expert in the area of third-party monitoring of humanitarian aid in Yemen.

^13^(Arabic) http://yemeninews.net/show2613047.html (accessed August 15, 2023).

^14^https://dhsprogram.com/pubs/pdf/fr296/fr296.pdf (accessed August 15, 2023).

^15^Interview with woman- academic professor in University of Sana'a Yemen.

^16^Interview with man- research analyst working in research center- Yemen.

^17^Interview with woman-researcher in the area of Internally Displaced Persons and Incarcerated Persons.

^18^ibid.

^19^ibid.

^20^Interview with man- human rights field monitor.

^21^ibid.

^22^Interview with man-field data collector in rural Yemen.

^23^Interview with woman-former researcher with Women National Committee in Yemen.

^24^Interview with man- human rights field monitor.

^25^Interview with man-field data collector in rural Yemen.

^26^Also known as Ayb aswad—(black shame) is a tribal tradition which shames any act of assault on women especially during conflict. This law protects women and places severe consequences if they are kidnapped, injured or killed during conflict and enables them to perform tasks which men in the same position are not safe to do.

^27^Interview with man- human rights field monitor.

^28^Interview with woman- director of local NGO providing GBV services.

^29^Ibid.

^30^Interview with woman researcher in the area of Internally Displaced Persons and Incarcerated Persons.

^31^ibid.

^32^UN Women. https://interactive.unwomen.org/multimedia/infographic/humanitarianaction/en/index.html (accessed October 3, 2023).

^33^Interview with research analyst with an established research center in Yemen.

^34^Interview with former researcher with Women National Committee in Yemen.

^35^Interview with international expert in the area of third-party monitoring of humanitarian aid in Yemen.

^36^Ibid.

^37^Interview with woman-feminist/activist.

^38^Interview with man-independent researcher.

^39^Interview with international expert in the area of third-party monitoring of humanitarian aid in Yemen.

^40^Interview with man human rights field monitor in north Yemen.
